# First case of amebic liver abscess 22 years after the first occurrence

**DOI:** 10.1051/parasite/2015020

**Published:** 2015-06-18

**Authors:** Benoît Nespola, Valérie Betz, Julie Brunet, Jean-Charles Gagnard, Yves Krummel, Yves Hansmann, Thierry Hannedouche, Daniel Christmann, Alexander W. Pfaff, Denis Filisetti, Bernard Pesson, Ahmed Abou-Bacar, Ermanno Candolfi

**Affiliations:** 1 Laboratoire de Parasitologie et de Mycologie Médicale, Hôpitaux Universitaires de Strasbourg 67091 Strasbourg France; 2 Service de Néphrologie et Hémodialyse, Hôpitaux Universitaires de Strasbourg 67091 Strasbourg France; 3 Institut de Parasitologie et de Pathologie Tropicale, Université de Strasbourg 67091 Strasbourg France; 4 Service de Maladies Infectieuses et Tropicales, Hôpitaux Universitaires de Strasbourg 67091 Strasbourg France; 5 Service de Médecine A, Centre Hospitalier de Sélestat 67600 Sélestat France

**Keywords:** *Entamoeba histolytica*, Liver abscess, Laboratory diagnosis, Serology, PCR

## Abstract

A 72-year-old man consulted in November 2012 for abdominal pain in the right upper quadrant. The patient had a history of suspected hepatic amebiasis treated in Senegal in 1985 and has not traveled to endemic areas since 1990. Abdominal CT scan revealed a liver abscess. At first, no parasitological tests were performed and the patient was treated with broad-spectrum antibiotics. Only after failure of this therapy, serology and PCR performed after liver abscess puncture established the diagnosis of hepatic amebiasis. The patient was treated with metronidazole and tiliquinol-tilbroquinol. Amebic liver abscess is the most frequent extra-intestinal manifestation. Hepatic amebiasis 22 years after the last visit to an endemic area is exceptional and raises questions on the mechanisms of latency and recurrence of these intestinal protozoan parasites.

## Abstract (fr)

Un homme de 72 ans consulte en novembre 2012 pour des douleurs abdominales dans le quadrant supérieur droit. Le patient présente un antécédent probable d’abcès amibien du foie traité en 1985 au Sénégal et n’a pas voyagé en région d’endémie depuis 1990. Le scanner abdominal réalisé met en évidence un abcès hépatique. Dans un premier temps, aucun examen parasitologique n’est effectué et le patient est traité par des antibiotiques à large spectre. Suite à l’échec de ce traitement, la sérologie et la PCR réalisées après ponction de l’abcès hépatique, établissent le diagnostic d’amibiase hépatique. Le patient est traité par métronidazole et tiliquinol-tilbroquinol. L’abcès amibien du foie est la manifestation extra-intestinale de l’amibiase la plus fréquente. L’amibiase hépatique 22 ans après le dernier voyage en zone d’endémie est exceptionnelle et soulève des questions concernant les mécanismes de latence et de récurrence des protozoaires intestinaux.

## Introduction

*Entamoeba histolytica*, an amebozoan parasite specific to humans, is the causative agent of human amebiasis, endemic in most tropical and subtropical countries. Amebic colitis and liver abscess (ALA) are the most frequent intestinal and extra-intestinal manifestations [25]. The majority of patients develop ALA within 5 months following travel to endemic areas, but some cases with a prolonged latency period have been described (up to 32 years) [11, 28].

*E. histolytica* infection occurs when mature cysts are ingested, mainly from fecally contaminated water and/or food, most frequently in the developing world. ALA is caused by hematogenous spread of trophozoites from intestinal mucosa to the liver through the portal vein. Even though various organs, such as the brain, liver, and lungs, can be affected by extra-intestinal amebiasis, liver abscess is the most frequent manifestation. The clinical symptoms of ALA include fever, weight loss, dull and aching abdominal pain in the right upper quadrant and hepatomegaly [2, 29]. Rupture of abscess and dissemination in the pleural, peritoneal, or pericardial cavities are the major complications [25]. Diagnosis of ALA is typically based on the clinical symptoms, characteristic of radiological imaging and serology. Diagnosis can be confirmed by PCR detection of *E. histolytica* DNA in the abscess fluid [29]. The general recommendation for treating invasive amebiasis is the combination of a tissue amebicide (principally metronidazole) with a luminal amebicide to eliminate any surviving parasites in the colon [9, 27]. Some cases of relapse of ALA have been described even with appropriate treatment [13, 21]. Despite its medical importance, there is a considerable lack of knowledge about the epidemiology of this infection. Forty million people are infected annually, although these estimations are skewed by the inclusion of the morphologically identical but non-pathogenic species *Entamoeba dispar*. *E. histolytica* causes up to 100,000 deaths per year, placing amebiasis second only to malaria in terms of mortality due to protozoan parasites [26]. France is not an endemic region for amebiasis, but sporadic cases of locally acquired infections have been reported [1, 15, 16]. Here, we report a case of amebic liver abscess relapse 22 years after the first occurrence and without any travel to endemic areas.

## Case presentation

A 72-year-old man, residing in Eastern France (Alsace), was admitted to the medical intensive care unit in October 2012 for acute calculous cholecystitis associated with severe sepsis. No liver abscess was diagnosed at that time. He had a history of suspected amebic liver abscess, operated and treated by metronidazole, after a trip to Senegal in 1985. The last time the patient traveled to endemic areas dates back to 1990, i.e. the West Indies. In November 2012, a few days after his release from the hospital, the patient consulted again for abdominal pain in the right upper quadrant with an inflammatory syndrome (C-reactive protein: 237 mg/L, procalcitonin: 71.2 ng/mL) without fever. Abdominal CT scan revealed a liver abscess. A liver puncture sample sent to the bacteriology laboratory was sterile. No parasitological sampling was conducted. A broad-spectrum antibiotic treatment by vancomycin (2 g/day), Tazocin (piperacillin/tazobactam) (12 g/day), and metronidazole (1500 mg/day) was implemented for a period of 3 weeks. Treatment with metronidazole was not continued by the patient due to poor digestive tolerance.

In December 2012, the patient was again hospitalized in the nephrology department for acute renal failure probably related to drug toxicity due to trimethoprim/sulfamethoxazole (creatinine: 360.4 μmol/L, urea: 14.3 mmol/L, Glomerular Filtration Rate: 15 ml/min/1.73 m^2^).

**Figure 1.**
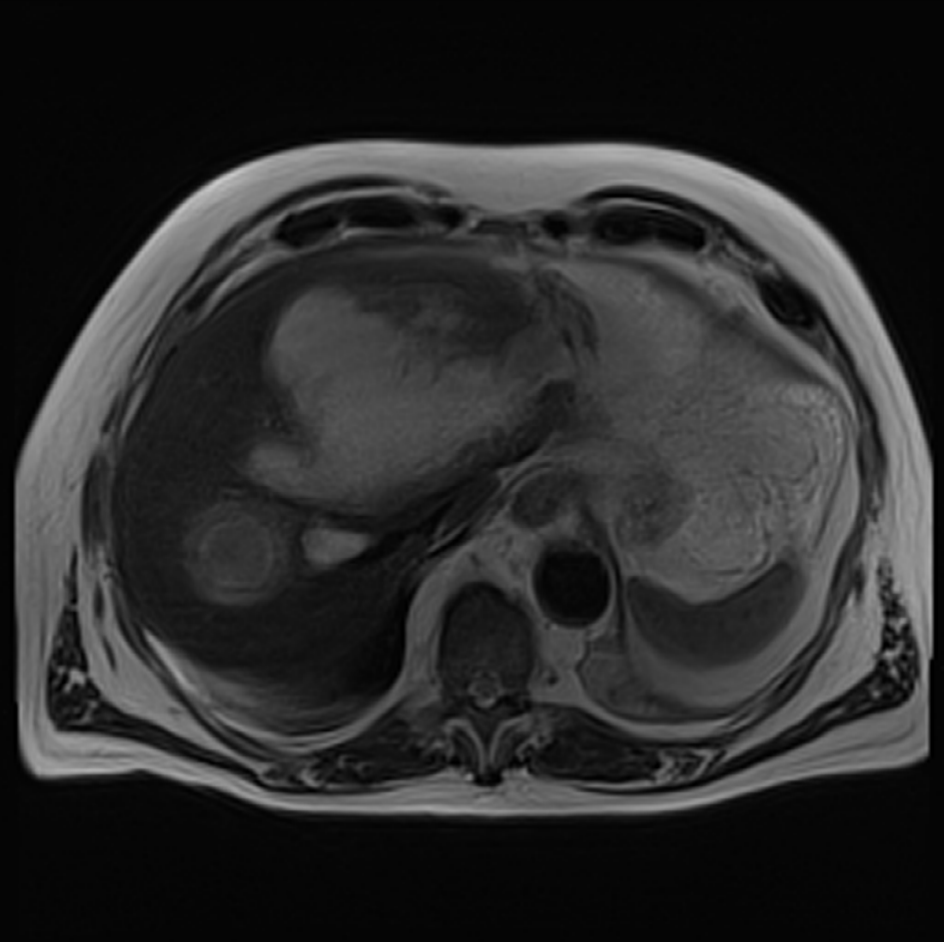
Axial T2 weighted magnetic resonance cholangiopancreatography (MRC) images showing a voluminous and heterogeneous collection in the left liver lobe (amoebic abscess).

**Figure 2.**
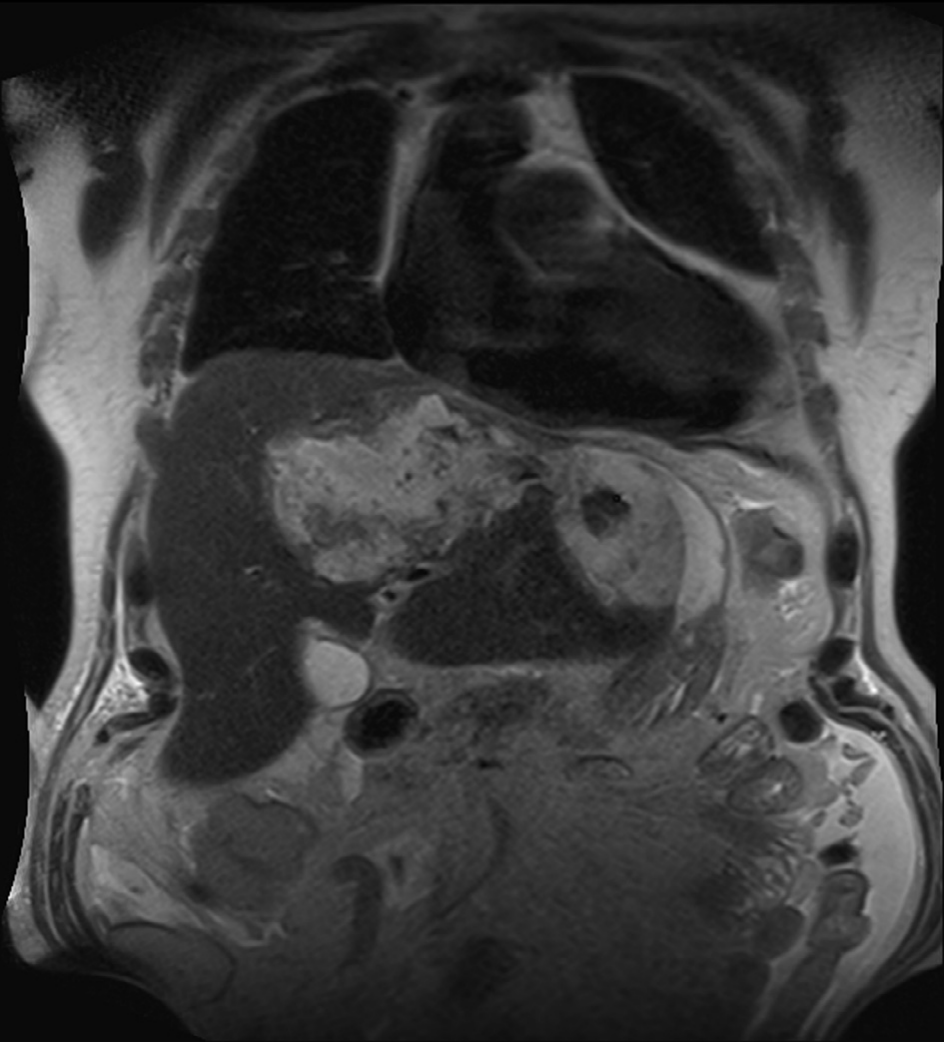
Coronal T2 weighted MRC images showing a voluminous and heterogeneous collection in the left liver lobe (amoebic abscess).

**Figure 3.**
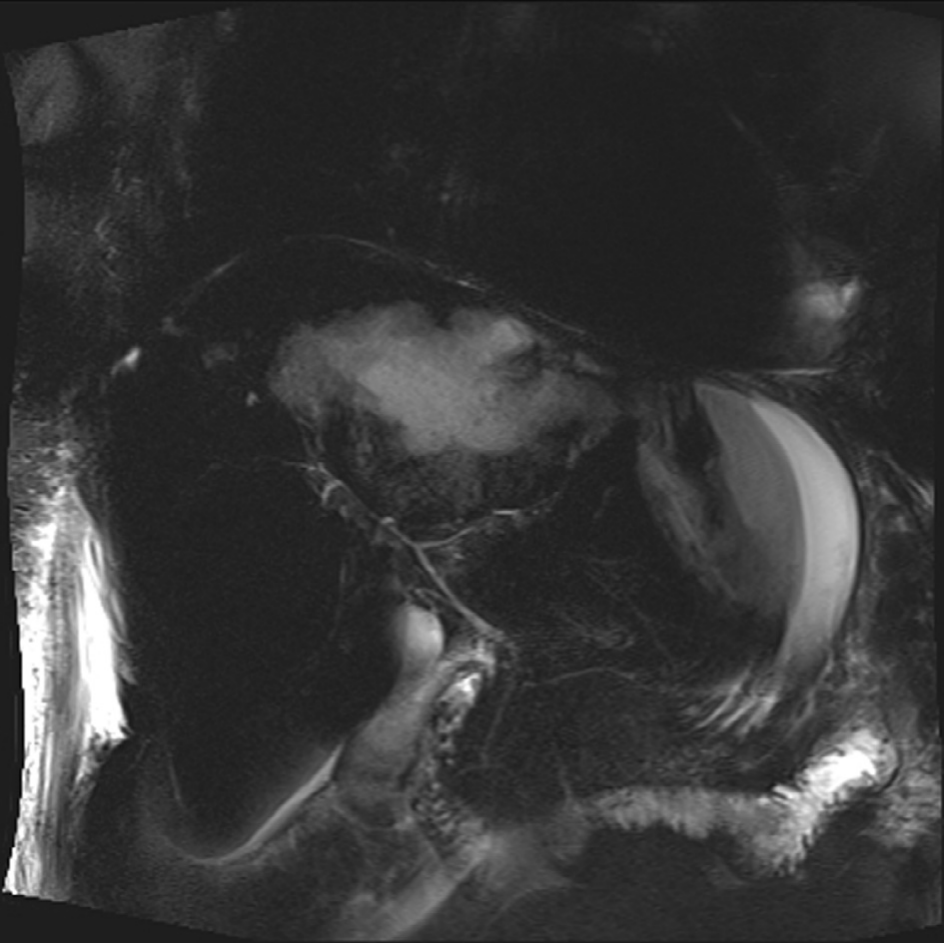
Three-dimensional (3D) MRC of the patient showing no bile duct dilatation.

At this time, the patient presented no biological inflammatory syndrome (CRP: 7.6 mg/L) or liver anomalies (ASAT: 19 U/L, ALAT: 13 U/L, gammaGT: 47 U/L, PAL: 154 U/L, total bilirubin: 7.4 μmol/L, conjugated bilirubin: 3.6 μmol/L) and was apyretic. MR cholangiopancreatography revealed a partitioned collection fluid (6.6 cm × 4 cm) in segments I and II, and an abscess in segment VII (5.5 cm × 5 cm) (Figs. 1–3).

Hydatid disease and alveolar echinococcosis serologies (these larval cestodes being endemic in Eastern France) were negative. However, amebiasis serology by ELISA Ridascreen *E. histolytica* IgG, (R-Biopharm GmbH, Darmstadt, Germany) was positive (IgG: 8.72; threshold: 0.9), as was latex agglutination (Bichro-Latex Amibe Fumouze, Fumouze Diagnostics, Levallois-Perret, France). In-house *Entamoeba histolytica* PCR was performed on a puncture of the liver abscess. DNA was extracted from 200 μL of the sample using the QIAamp DNA Mini Kit (Qiagen, Courtaboeuf, France). PCR amplification was carried out as described by Gonin and Trudel [8] using HotStarTaq DNA Polymerase PCR Buffer 1X (Qiagen), 1 U HotStarTaq DNA Polymerase (Qiagen), 200 μM dNTPs, 1 mM MgCl2, and 0.5 μM of each primer ED1 + EDH2 for the detection of *Entamoeba dispar* or EH1 + EDH2 for the detection of *Entamoeba histolytica*. Cycling conditions were as follows: 15 min incubation at 95 °C followed by 40 cycles consisting of 30 s at 95 °C, 60 s at 51 °C, and 40 s at 72 °C, with a final 5 min elongation at 72 °C. The PCR was monitored by positive and negative controls. This in-house PCR test was positive, thus confirming the diagnosis of ALA.

Direct examination by microscopy and culture of the aspirated liver abscess fluid were negative. The stool examination performed in this patient in January 2013 was also negative. The clinical course was favorable after 14 days of metronidazole and 10 days of tiliquinol-tilbroquinol.

## Discussion

Amebiasis occurs in 10% of the world’s population and is most common in tropical and subtropical regions. ALA is the most common extra-intestinal manifestation of amebiasis. ALA develops in less than 1% of patients infected with *E. histolytica*. The disease should be suspected in anyone with a history of residency in or travel to an endemic area associated with fever, right upper quadrant pain, and significant hepatic tenderness [22]. In Europe, ALA is observed in European-born travelers visiting endemic countries and in foreign-born patients living in Europe. In France, a retrospective analysis between 2002 and 2006 in the area of Paris reported 331 patients with positive amebiasis serology. Among these patients, 30.8% had amebic liver abscesses; 45.5% were European-born patients [4].

For adequate clinical management, it is important to rapidly diagnose ALA and to distinguish it from other, particularly bacterial, causes of liver abscesses. This can be challenging, as clinical and imaging findings are similar for amebic and pyogenic liver abscesses, necrotic hepatoma or echinococcal cyst, and therefore have poor specificity [5, 18]. Stool microscopy in cases of ALA is generally negative. Moreover, it is impossible to differentiate the pathogenic species (*E. histolytica*) from non-pathogenic species (*E. dispar* or *E. moshkovskii*), except if phagocytized red blood cells are present within the trophozoite, thus confirming the diagnosis of *E. histolytica*. Diagnosis of ALA is usually based on amebiasis serology even though negative serology cases have been described [17, 20]. The combination of serological tests and PCR on a puncture of the liver abscess offers the best diagnostic approach.

Two hypotheses can be advanced in order to explain this liver abscess in our patient: new contamination or persistence of endogenous abscess causing a very late relapse.

The hypothesis of recontamination cannot be excluded although our patient had not traveled to endemic areas since 1990. The current mobility of people around the world favors the spread of many diseases to non-endemic countries. It also promotes the emergence of these diseases around imported cases in persons who have not themselves traveled outside their own country. In an early series of 152 cases of hepatic amebiasis in France, there were eight cases of autochthonous infection [14]. In a more recent retrospective study involving 20 patients, one case was attributed to autochthonous infection [6]. Moreover, the hypothesis of transmission by oral-anal sex cannot be discarded even though the interview was not conducted in this direction [7, 12].

Secondly, the assumption of asymptomatic persistence of *E. histolytica* in the digestive tract for many years is to be considered. Indeed, there is no trace of treatment by tiliquinol-tilbroquinol in the patient’s medical record for his first suspected liver abscess in 1985. Two similar cases were recently published. In 2012, Singal et al. described a patient living in India (an endemic area) with multiple relapses despite treatment with metronidazole [24]. In the other article, the authors describe a patient living in France with a recurrent amebic abscess 10 years after the first occurrence [10]. In these two cases, patients had not received tiliquinol-tilbroquinol. This illustrates the importance of combination therapy with tissue and contact amebicides in order to eliminate any intestinal colonization by *E. histolytica*. This second hypothesis seems to be the most likely, due to the absence of travel to an endemic area and the low risk of acquiring the infection in France.

No drug resistance has been described to current amebicidal agents [3]. However, cases of relapse without travel in endemic areas have been described in patients with adequate amebicidal agents. Relapses of ALA are rare and have been estimated at 0.004% of the ALA patients per year. A long latent period may exist between the first episode of ALA and the relapse (up to 17 years in a case described by Shizuma et al., 2000) [10, 13, 19, 21, 23, 24].

Recurrence of hepatic amebiasis 22 years after the last visit to an endemic area is exceptional, but it should always be kept in mind and confirmed by amebic serology and PCR in a patient with a liver abscess, even in the absence of a recent stay in an endemic area.
